# Overcoming Low‐Field Limitations: High‐Quality *Ex Vivo* Soft Tissue Imaging with Compact MRI Systems

**DOI:** 10.1002/cpz1.70288

**Published:** 2026-01-02

**Authors:** Andrea Litwak, Andrea Sarabia, Mikayla Tamboline, Shumpei Mori, Shili Xu

**Affiliations:** ^1^ Department of Molecular and Medical Pharmacology University of California Los Angeles Los Angeles California; ^2^ Crump Institute for Molecular Imaging University of California Los Angeles Los Angeles California; ^3^ David Geffen School of Medicine University of California Los Angeles Los Angeles California; ^4^ Department of Medicine University of California Los Angeles Los Angeles California; ^5^ Jonsson Comprehensive Cancer Center, David Geffen School of Medicine University of California Los Angeles California; ^6^ These authors contributed equally to this work

**Keywords:** *ex vivo* tissue samples, permanent magnet, preclinical low‐field MRI, tissue preparation

## Abstract

High‐field magnetic resonance imaging (MRI) scanners have been the primary choice for high‐quality soft tissue imaging, but use remains limited by high costs, complex siting requirements, and infrastructure demands. In contrast, preclinical MRI scanners with a low‐field permanent magnet are increasingly being adopted in research due to their lower cost, portability, and ease of installation. However, their reduced intrinsic signal‐to‐noise ratio (SNR) and limited pulse sequence availability constrain their use for high‐quality *ex vivo* soft tissue characterization. To overcome these limitations, we established an optimized imaging protocol using a compact 1‐Tesla permanent‐magnet preclinical MRI system for high‐quality *ex vivo* soft tissue imaging. A 25‐mm (length) × 23‐mm (diameter) coil was employed to image both human and animal soft tissue samples. Protocol optimization focused on four key parameters, namely the number of excitations (NEX), repetition time (TR), echo time (TE), and slice thickness, using a 3D gradient‐echo (3D‐GRE) acquisition sequence. Increasing the NEX enhances the SNR through signal averaging, extending the TR further improves the SNR, reducing the TE provides improved tissue contrast, and thinner slices improve image resolution. A typical 3D‐GRE MRI scan using the imaging protocol takes 13 hr. The approach also incorporates standardized *ex vivo* sample preparation, including staining with gadolinium diethylenetriaminepentaacetic acid to enhance tissue contrast, and background proton signal suppression and sample immobilization with Fluorinert or agarose. Collectively, these optimizations substantially improve the performance of low‐field permanent‐magnet MRI scanners for high‐quality soft tissue imaging. This set of protocols provides a practical framework for researchers to expand the capabilities of low‐field MRI systems, enabling more widespread access to advanced tissue characterization methods that support disease research and therapeutic development. © 2026 The Author(s). *Current Protocols* published by Wiley Periodicals LLC.

**Basic Protocol 1**: Tissue sample preparation with Gd‐DTPA and Fluorinert

**Alternate Protocol**: Tissue sample preparation with Gd‐DTPA and agarose

**Basic Protocol 2**: High‐quality sample imaging with 1‐Tesla compact MRI

## INTRODUCTION

Magnetic resonance imaging (MRI) is an essential tool in both clinical disease diagnosis and preclinical research for noninvasive visualization of tissue structure and pathology. High‐field MRI systems, typically operating at ≥7 Tesla in preclinical settings, provide superior image resolution and an improved signal‐to‐noise ratio (SNR), making them particularly valuable for detailed characterization of *ex vivo* soft tissue samples (Ladd et al., 2018). These systems enable visualization of fine structural features, identification of microarchitectural changes, and improved assessment of pathological processes. However, high‐field systems are costly, require complex infrastructure, and present significant siting and operational challenges, which limit their availability to many research groups (Shaffer et al., 2022).

In contrast, low‐field MRI systems based on permanent magnets (≤1 Tesla) are more affordable, compact, and portable and have simpler siting requirements (Tempel‐Brami et al., 2015). These features have driven their widespread adoption in preclinical research laboratories, particularly for routine *in vivo* small‐animal imaging (Arnold et al., 2023). However, low‐field MRI systems suffer from a reduced intrinsic SNR and limited pulse sequence availability (Ayde et al., 2025), and their default imaging protocols are optimized for short scan times to minimize motion artifacts in live animals and maximize animal safety. As a result, these systems often fall short in delivering the high spatial resolution, high SNR, and high contrast required for detailed *ex vivo* soft tissue characterization.

Optimizing sample preparation and acquisition parameters provides an opportunity to overcome these inherent limitations of low‐field MRI. The quality of MRI images depends on multiple technical factors, including magnetic field strength, coil geometry, gradient performance, voxel size, and acquisition sequence design. For low‐field systems, careful adjustment of parameters such as the repetition time (TR), echo time (TE), and number of excitations (NEX) can substantially enhance both the SNR and tissue contrast. In addition, sample preparation, including with the use of gadolinium‐based contrast agents, background proton signal suppression, and immobilization techniques, can improve image quality by enhancing tissue delineation and minimizing motion artifacts.

Here, we describe an optimized set of protocols for high‐quality *ex vivo* soft tissue imaging using a compact 1‐Tesla MRI system with a permanent magnet. Using a 25 × 23–mm coil and a 3D gradient‐echo (3D‐GRE) sequence, we systematically adjusted the NEX, TR, TE, and slice thickness to improve the SNR, contrast, and spatial resolution. A typical MRI scan using the optimized imaging protocol (Basic Protocol 2) on a 1‐Tesla scanner takes 13 hr. The article also incorporates standardized tissue preparation with gadolinium diethylenetriaminepentaacetic acid (Gd‐DTPA) staining and background proton signal suppression and sample immobilization with Fluorinert (Basic Protocol 1) or agarose (Alternate Protocol), providing enhanced structural clarity and reproducibility.

To our knowledge, prior optimization studies have primarily focused on high‐field (≥7‐Tesla) MRI systems (Blocker et al., 2021; Hanalioglu et al., 2024; JuanYin et al., 2009; Remus et al., 2019; Shapiro et al., 2004), with limited attention to 1‐Tesla scanners with a permanent magnet. Our work addresses this gap by presenting a novel, detailed set of protocols for high‐quality *ex vivo* soft tissue imaging on a low‐field, permanent‐magnet MRI system. This method enables more widespread access to advanced tissue characterization in laboratories equipped with compact low‐field MRI systems, thereby expanding opportunities for biomedical research and therapeutic development.

*NOTE*: All protocols involving animals must be reviewed and approved by the appropriate Animal Care and Use Committee and must follow regulations for the care and use of laboratory animals.

## Basic Protocol 1 TISSUE SAMPLE PREPARATION WITH Gd‐DTPA AND FLUORINERT

The aim of this protocol is to prepare an *ex vivo* soft tissue sample for MRI imaging. Sample embedding in Fluorinert (this protocol) or agarose (Alternate Protocol) helps the sample to remain immobile during imaging, which is crucial for obtaining optimal images. This method describes sample embedding in Fluorinert, a perfluorinated compound without “movable protons,” yielding the highest MRI image quality, with clear and defined structural details and minimal background noise. Fluorinert also provides increased flexibility and allows the *ex vivo* tissue sample to be manipulated and transferred multiple times following scanning. However, if the cost of Fluorinert becomes a concern for a large study and increased background noise is acceptable, tissue samples can be embedded in 5% agarose gel instead of Fluorinert, which is detailed in the Alternate Protocol.

This protocol consists of four sections: (1) obtaining and preparing *ex vivo* tissue samples, (2) soaking in contrast agent, (3) embedding in Fluorinert, and (4) storing the samples for future use. The first section describes first perfusing the animal to improve sample quality when the animal is dissected. *Ex vivo* tissue samples are then soaked in Gd‐DTPA overnight. Prior to MRI, the Gd‐DTPA‐soaked samples are embedded in Fluorinert to minimize background noise and prevent any movement.

### Materials

C57BL/6J mice (The Jackson Laboratory, cat. no. 000664)
Isoflurane (McKesson Medical‐Surgical, cat. no. 803250)
Oxygen
10 U/ml heparinized saline [heparin (McKesson, cat. no. 63739‐931‐14; normal saline (Vedco, cat. no. VINV‐SALN‐1000)]
10% (v/v) formalin (Fisher Scientific, cat. no. SF98‐4)
70% (v/v) ethanol (Fisher Scientific, cat. no. BP82011)
Phosphate‐buffered saline (PBS; pH 7.4; Gibco, Fisher Scientific, cat. no. 10010001)
1 mM Gd‐DTPA (see recipe)
Fluorinert FC‐70 (Millipore Sigma, cat. no. F9880‐25 ml)

Anesthesia chamber
Isoflurane vaporizer (Stoelting^™^, Fisher Scientific, cat. no. 10‐000‐928)
Nose‐cone system
20‐ml syringes (Becton Dickinson, cat. no. 302830)
Infusion needles (syringe needle, gauge 27, Becton Dickinson, cat. no. 305109)
Foam dissection bed
Pins
Fine‐tip dissecting forceps (Fisher Scientific, cat. no. 08‐953G)
Fine scissors (Fisher Scientific, cat. no. 17‐467‐109)
Paper towels
5‐ml syringes (Becton Dickinson, cat. no. 309646)
1‐ml insulin syringes (EXEL Medical Products, cat. no. 26027)
Transfer pipets
Hot glue gun (Stanley, cat. no. GR25)
Parafilm (Parafilm, cat. no. PM999)

*NOTE*: Please follow reagents’ safety data sheets and work with institutional safety teams for handling and disposal of reagents.

#### Obtain and prepare ex vivo tissue samples

1 Anesthetize the animal:
  a. Place the C57BL/6J mouse inside an anesthesia chamber. Use a mixture of isoflurane and oxygen gas at 2.0% concentration to anesthetize mouse using an isoflurane vaporizer.
  b. Keep the animal under anesthesia for ∼5 min, or until its breathing noticeably slows (Taschereau et al., 2025).
    Assess the depth of anesthesia by firmly pinching the webbing between the toes to test for the toe pinch reflex. Proper anesthesia depth is indicated by the absence of a toe‐pinch reflex.
  c. Once fully anesthetized, remove the animal from the chamber and immediately transfer it to a nose‐cone system to continue anesthesia delivery during perfusion.
2 Perform transcardiac perfusion with heparinized saline:
  a. Fill a 20‐ml syringe with heparinized saline (10 U/ml) and connect the infusion needle.
  b. Expel any trapped air from the syringe and infusion needle by gently pressing the plunger until saline appears at the tip.
  c. Place the mouse face up on a foam dissection bed.
  d. Extend and pin all four limbs outward to prevent movement during perfusion.
  e. Using fine‐tip dissecting forceps, grasp and elevate the skin over the chest.
  f. Using fine scissors, make an incision along the thoracic cavity to expose the heart.
  g. Carefully insert the needle filled with heparinized saline into the left ventricle at an angle parallel to the heart.
    Do not insert the needle too deeply to prevent it from perforating the left atrium or right ventricle.
  h. Begin perfusion with heparinized saline at room temperature (1 ml/5 s).
  i. Continue until target tissues appear pale and effluent runs clear.
3 Collect organs of interest and immediately place them into 10% formalin for 24 hr.
4 After 24 hr in 10% formalin, place the sample in 70% ethanol for storage (de Guzman et al., 2016).
  Formalin can cause tissue contraction, which should be considered in anatomical analysis (Brizzi et al., 1994). Tissues in 70% ethanol may be stored at room temperature. It is recommended to image the sample before the fifth month of storage is complete (de Guzman et al., 2016).

#### Soak in contrast agent (Gd‐DTPA)

**Figure 1:**
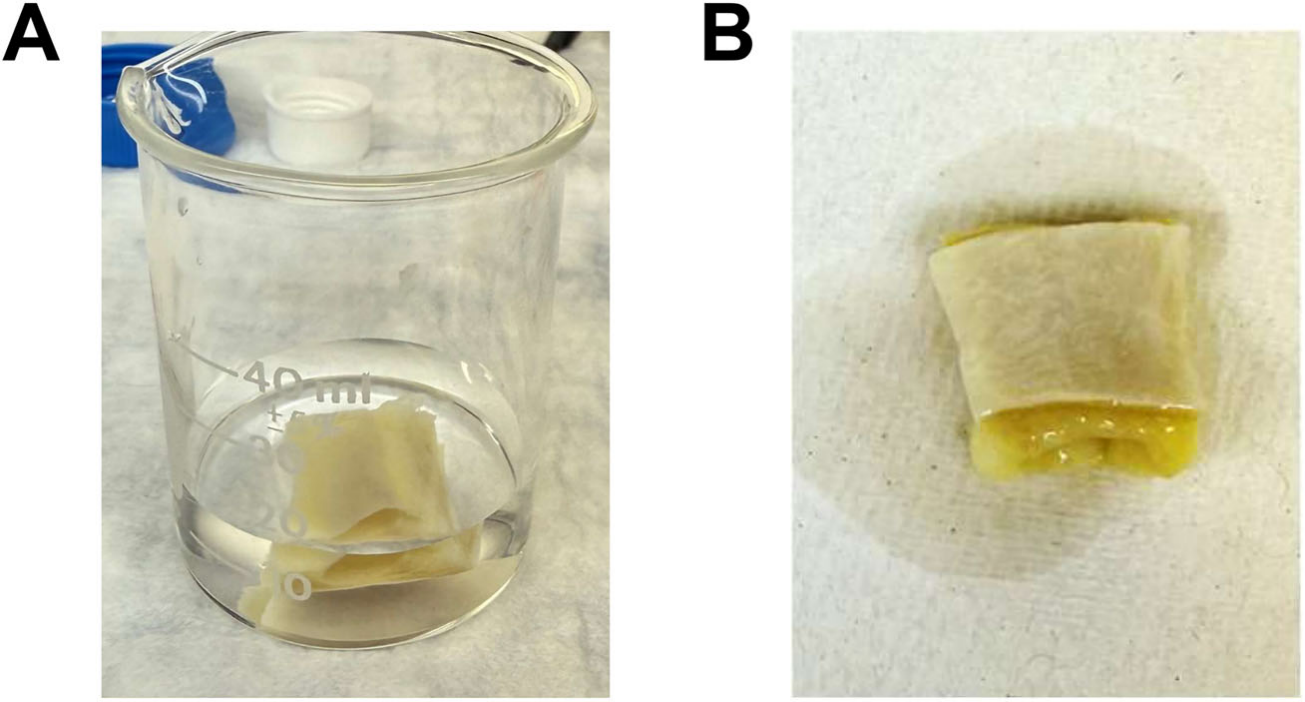
Rinsing and drying tissue samples. A tissue sample is (**A**) rinsed with PBS and then (**B**) dried with a paper towel.

5 Between 24 and 72 hr before MRI scanning, remove organs/tissue samples from 70% ethanol and rinse them with PBS (Fig. 1A).

6 Pat them dry with a paper towel (Fig. 1B).
7 Place organs in 1 mM Gd‐DTPA (1 mg Gd‐DTPA per 1.826 ml PBS) overnight at room temperature (Shapiro et al., 2006).

#### Embed samples in Fluorinert

**Figure 2:**
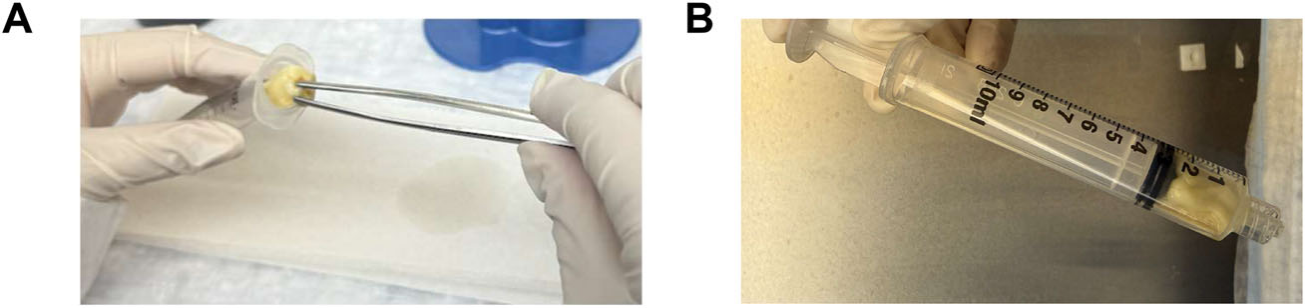
Securing the tissue sample in a syringe. (**A**) Remove the plunger from the syringe and place the tissue sample inside. (**B**) Reinsert the plunger and secure the sample.

8 Remove samples from 1 mM Gd‐DTPA solution and rinse with PBS three times (to remove any excess contrast agent from surface crevices).
9 Remove the plunger from a 5‐ml syringe.
10 Place a sample inside the 5‐ml syringe (Fig. 2A).

**Figure 3:**
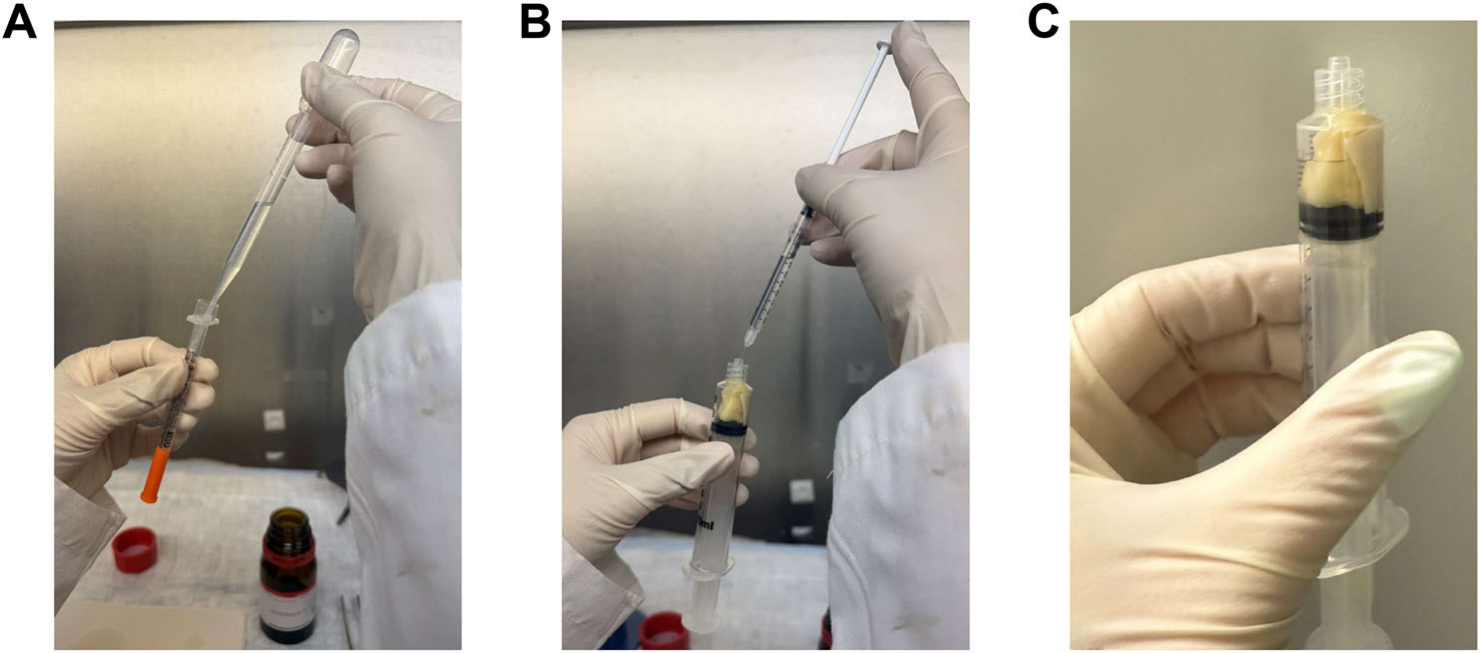
Embedding the tissue sample in Fluorinert. (**A**) Fill an insulin syringe with Fluorinert using a transfer pipet. (**B**) Inject Fluorinert into the sample‐containing 5‐ml syringe. (**C**) Fill the sample compartment with Fluorinert until no air remains.

11 Reinsert plunger into the syringe barrel and gently push plunger forward until the sample is secured against the tip of the syringe without any movement (Fig. 2B). Take caution not to press the sample excessively to avoid changing the internal structure of the tissue.
12 Fill a 1‐ml insulin syringe with Fluorinert FC‐70 using a transfer pipet (Fig. 3A).

**Figure 4:**
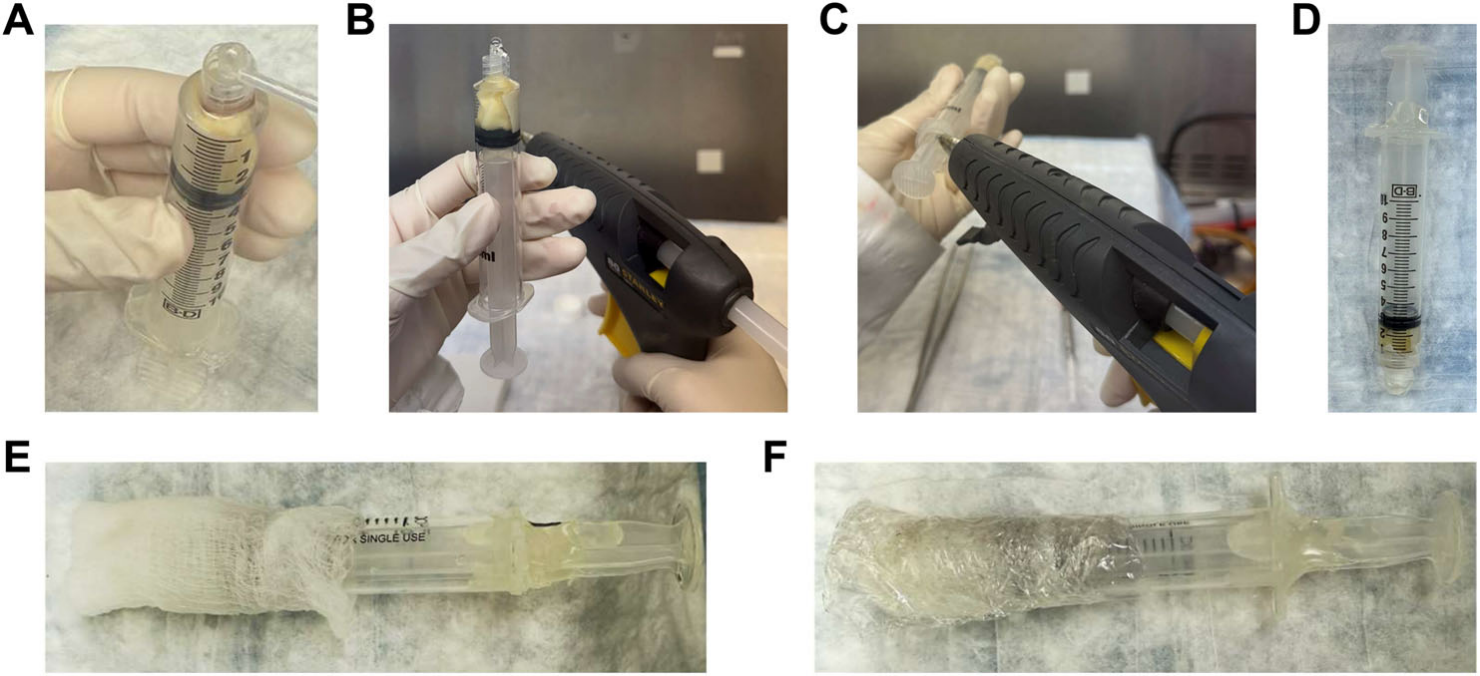
Sealing the sample‐containing syringe. (**A**) Fill the outer area surrounding the needle insertion opening with water. Seal (**B**) the syringe opening and (**C**) the plunger gaps with hot glue. (**D**) Place a piece of Parafilm over the syringe opening. (**E**) Wrap the syringe with a piece of wet gauze. (**F**) Wrap a piece of laboratory wrap over the wet gauze.

13 Inject Fluorinert into the 5‐ml syringe containing the sample through the needle insertion opening (Fig. 3B).
14 Repeat steps 12 and 13 to fill the 5‐ml syringe with Fluorinert until the sample is fully embedded and there are no air bubbles visible within the 5‐ml syringe (Fig. 3C).
15 Fill the outer area surrounding the needle insertion opening with water (Fig. 4A), ensuring that water does not enter the syringe, where the Fluorinert and sample are.
  For preclinical low‐field MRI scanners that perform coil calibration during long scans, small samples with low levels of movable protons could cause error in these coil calibrations, resulting in failure of a long scan. This step ensures that there are enough movable protons within the MRI's field of view (FOV) for coil calibrations during long scans.

16 Seal the sample‐containing 5‐ml syringe's opening with hot glue using a hot glue gun (Fig. 4B).
17 Hot‐glue the syringe plunger to prevent it from moving (Fig. 4C).
18 Wrap the syringe with a piece of Parafilm (Fig. 4D).
  If the MRI system has difficulty during calibration, particularly with small tissues, wrap wet gauze around the syringe (Fig. 4E) and seal it with laboratory wrap (Fig. 4F).

#### Store samples for future use

19 After MRI scanning (see Basic Protocol 2), remove samples from Fluorinert and rinse samples with PBS.
  Fluorinert can be saved and reused for future samples.
20 Place samples back into 70% ethanol for storage.
  Alternatively, samples can be discarded.

## TISSUE SAMPLE PREPARATION WITH Gd‐DTPA AND AGAROSE

This method offers an alternative to embedding the sample in Fluorinert, as described in Basic Protocol 1. This protocol describes sample embedding in 5% agarose gel, which provides a cost‐effective and efficient method to immobilize the sample for imaging. Because agarose gel contains “movable protons”, this method will result in higher background noise compared to the use of Fluorinert. Once the sample is embedded in 5% agarose gel, it can be stored and re‐imaged multiple times. However, compared to the sample embedding with Fluorinert (Basic Protocol 1), it may be difficult to remove the sample from the agarose once embedded, which could result in damage to the sample or residual agarose being left on the sample.

### Materials

PBS (pH 7.4; Gibco, Fisher Scientific, cat. no. 10010001)
Agarose, biological grade (Thermo Scientific, cat. no. J32802‐22)

50‐ml glass beaker
Microwave
Glass stirring rod
15‐ml conical tubes (Fisher Scientific, cat. no. 14‐959‐53A)
Tweezers
Parafilm (Parafilm, cat. no. PM999)

Additional reagents and equipment for obtaining and preparing *ex vivo* tissue samples and soaking them in the contrast agent Gd‐DTPA (see Basic Protocol 1, steps 1 to 7)

#### Prepare samples and soak in contrast agent

1 Follow Basic Protocol 1, steps 1 to 7, to obtain and prepare *ex vivo* tissue samples and soak them in the contrast agent Gd‐DTPA.

#### Embed samples in agarose gel

2 Remove samples from 1 mM Gd‐DTPA and rinse with PBS three times (to remove any excess contrast agent from unwanted crevices).
3 Prepare 5% (w/v) agarose by combining 10 ml PBS with every 0.5 g agarose powder in a 50‐ml glass beaker.
4 Microwave agarose mixture until it starts boiling.
5 Slowly stir the mixture with a glass stirring rod to avoid introducing bubbles into the hot agarose solution.
6 Pour a small amount of agarose solution into the 15‐ml conical tube, against the wall.
7 Before the agarose gel sets, gently lower a sample into the agarose solution using tweezers and hold the sample until the agarose gel cools down and solidifies.
8 If the remaining agarose solution in the glass beaker has begun to cool down, re‐microwave it. Fill the conical tube to the top and allow the poured agarose solution to cool down and solidify.
9 Re‐cap the conical tube and seal with Parafilm.

#### Store samples for future use

10 When an embedded sample is not in use, store it at 4°C.
  In addition to the Fluorinert method (Basic Protocol 1) and the agarose method described here for sample preparation, a potato starch suspension can also be used as a low‐cost, eco‐friendly alternative (Tsurugizawa et al., 2022, 2023; Wrightson et al., 2025). Potato starch is less flexible than Fluorinert in terms of sample preparation and adjustment for imaging, but it is far more affordable. Additionally, compared to agarose, potato starch does not increase the background noise for MRI.

## Basic Protocol 2 HIGH‐QUALITY SAMPLE IMAGING WITH 1‐TESLA COMPACT MRI

The aim of this protocol is to yield the highest imaging quality for *ex vivo* soft tissue samples. If the sample is prepared according to our optimized method and the parameters are adjusted accordingly, the user should obtain high‐quality images of the *ex vivo* tissue sample of interest.

### Materials

Syringe or conical tube containing sample (see Basic Protocol 1 and Alternate Protocol, respectively)

1‐Tesla MRI (Aspect M2 Compact High‐Performance MRI) and associated MRI acquisition software
25‐mm (length) × 23‐mm (diameter) coil
Paper tape

#### Turn on and set up the MRI system

**Figure 5:**
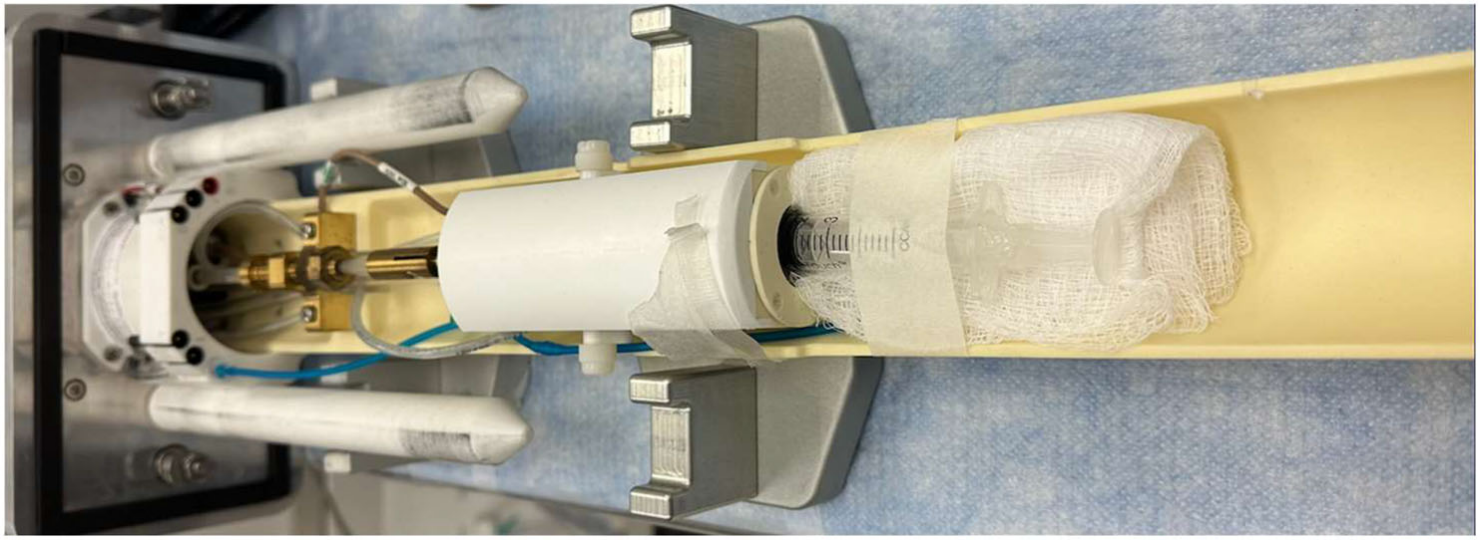
Securing the sample‐containing syringe to the 23‐mm‐diameter MRI coil. Gauze can be used to elevate the sample to the appropriate height, and paper tape is placed across the top of the syringe to prevent any movement.

1 Turn on the 1‐Tesla MRI machine hardware by pressing the power button.
*IMPORTANT NOTE*: It is imperative that the proper steps are followed to turn on the MRI machine and load the software. Failure to do so will interfere with the ability to initiate a scan properly.
2 Wait ≥1 min and then launch associated MRI acquisition software.
3 Place the tip of the syringe or the conical tube containing the sample into the 25‐mm (length) × 23‐mm (diameter) coil, ensuring the complete region of interest in the sample is inside the MRI's FOV (Fig. 5).
  Ensure that the syringe (see Basic Protocol 1) or the conical tube (see Alternate Protocol) lays parallel to the coil once inserted. If needed, elevate the syringe or the conical tube by placing folded 4 × 4–in. gauze underneath it.

4 Place a 4‐in. piece of paper tape over the syringe or the conical tube to secure the sample to the coil and prevent any unwanted movement.
5 Insert the coil into the MRI system.

#### Set up the MRI acquisition protocol and conduct imaging

6 Set up the acquisition protocol and perform imaging.
  MRI scans were conducted using a 1‐Tesla MRI system (Aspect M2, Aspect Imaging) equipped with a 25‐mm‐length × 23‐mm‐diameter mouse head coil. Coronal images were acquired using our optimized 3D‐GRE sequence with the following parameters: TR = 60 ms, TE = 5.97 ms, FOV = 37.11 mm × 37.11 mm, resolution = 0.1 mm × 0.1 mm × 0.2 mm (slice thickness), NEX = 70, and number of slices = 30 for samples in Fluorinert (40 for samples in agarose). Axial images were acquired using a 3D‐GRE sequence with the following parameters: TR = 60 ms, TE = 5.97 ms, FOV = 37.11 mm × 37.11 mm, resolution = 0.1 mm × 0.1 mm × 0.2 mm (slice thickness), NEX = 60, and number of slices = 30. An MRI scan with this protocol takes ∼13 hr. MRI images were visualized using AMIDE 1.0.5 (Loening & Gambhir, 2003).
  The quality of MRI imaging can be evaluated based on its SNR. Use $(\frac{mean}{standard\;deviation})\;$ of signal (sample) to noise (background) to calculate the SNR using AMIDE software or other DICOM viewers.

## REAGENTS AND SOLUTIONS

### Gd‐DTPA, 1 mM

1.826 ml PBS (pH 7.4; Gibco, Fisher Scientific, cat. no. 10010001)
1 mg gadolinium diethylenetriaminepentaacetic acid (Gd‐DTPA) powder (Med Chem Express, cat. no. HY‐P1001)
Store ≤3 months at 4°C

## COMMENTARY

### Background Information

**Figure 6:**
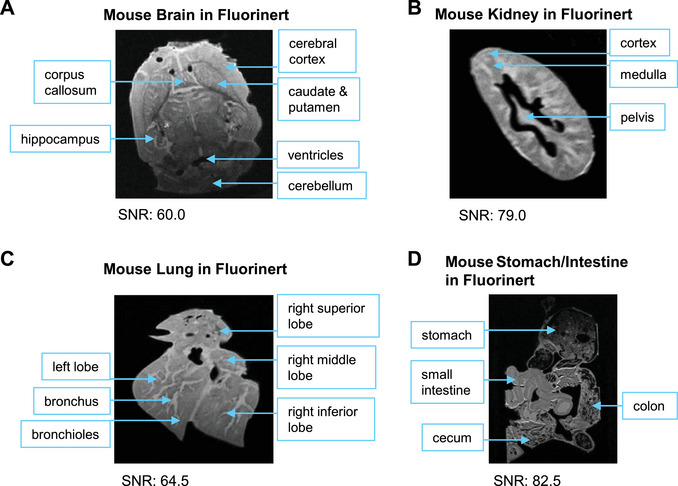
MRI images of *ex vivo* mouse tissues embedded in Fluorinert. MRI images of (**A**) a mouse brain (SNR: 60.0), (**B**) a mouse kidney (SNR: 79.0), (**C**) a mouse lung (SNR: 64.5), and (**D**) a mouse stomach and intestine (SNR: 82.5) are shown. Each of these scans took 13 hr.

**Figure 7:**
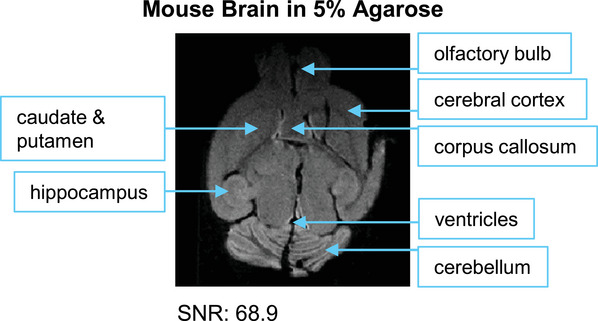
MRI image of an *ex vivo* mouse brain embedded in 5% agarose gel. Key anatomical structures, including the caudate and putamen, hippocampus, olfactory bulb, cerebral cortex, corpus callosum, ventricles, and cerebellum, can be visualized in the MRI image (SNR: 68.9). The scan time was 17.5 hr.

**Figure 8:**
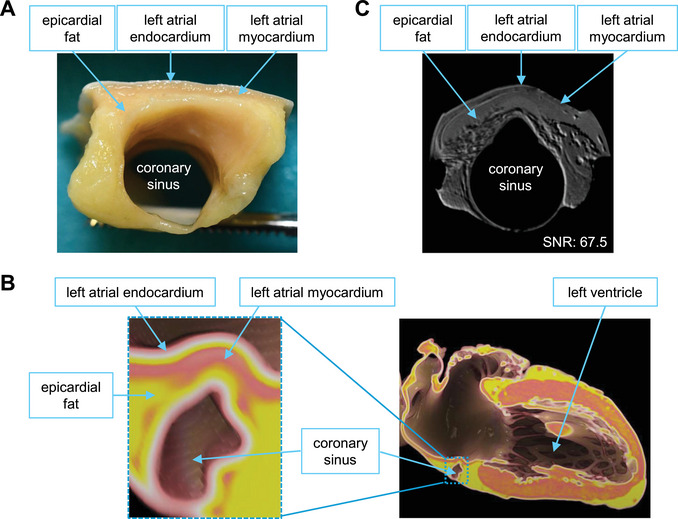
MRI image of an *ex vivo* human heart tissue. (**A**) Photograph of the human heart tissue. (**B**) Location of the tissue within the human heart. (**C**) MRI image of the human heart tissue embedded in Fluorinert. Detailed anatomical structures, including the epicardial fat, the left atrial endocardium, and the left atrial myocardium, can be visualized in the MRI image (SNR: 67.5). The scan time was 11.5 hr.

We developed this set of protocols given the lack of high‐quality *ex vivo* soft tissue MRI protocols, especially for 1‐Tesla preclinical MRI systems. The advantages of this approach are increased detailed structure visualization, spatial resolution, and contrast for low‐field MRI imaging. This approach has proven effective in imaging both *ex vivo* human and animal tissue samples. We have applied this set of protocols to the mouse brain (Fig. 6A), kidney (Fig. 6B), lung (Fig. 6C), and stomach and intestine (Fig. 6D) embedded in Fluorinert and the mouse brain embedded in agarose gel (Fig. 7). In addition, we also applied these protocols to human heart tissue (Fig. 8).

### Critical Parameters

#### Sample embedding

To ensure high quality of the MRI images, it is essential for the *ex vivo* tissue samples to remain completely immobilized during scanning while suspended in Fluorinert. A critical step in achieving this is the proper positioning of tissues inside the syringe in Basic Protocol 1. When placing the tissue sample in the syringe, a proper fit is vital. The tissue sample should not be squished against the syringe walls to prevent changes in the tissue structure. If the tissue sample is too large to fit inside a 5‐ml syringe, use a syringe of a larger size. The syringe plunger should be pressed firmly against the sample to prevent any sample movement, while taking care not to exert excessive pressure that could deform or compromise the structural integrity of the sample. Optimal compression maintains the sample position without introducing artifacts due to movement or deformation during scanning. Whereas Fluorinert (Basic Protocol 1) has been used for long scans as well as medium‐term storage between scans without reported detrimental effects (Roebroeck et al., 2019; Shatil et al., 2016), agarose (Alternate Protocol) use as a water‐based embedding medium could impact the relaxation properties of fixed tissues, and therefore, the period of the fixed tissue in agarose should be minimized (Vuckovic et al., 2020).

#### Contrast agent Gd‐DTPA

Gd‐DTPA (applied in Basic Protocol 1 and the Alternate Protocol) is a paramagnetic agent. The paramagnetic properties of Gd‐DTPA alter the spin state of the protons within the tissues. The protons are able to return to their relaxed state much sooner, allowing the radiofrequency (RF) pulse to be applied sooner.

#### MRI parameters

TR is a critical parameter because it determines the time between successive pulse sequences to the same slice, impacting the scan contrast and signal intensity. Longer TR values improve the SNR but increase scan time. Shorter TR values allow faster scan times but can lower the SNR. Optimizing TR is crucial to obtain an image with a balanced contrast to visualize structural details.

TE defines the time between the delivery of a RF pulse to the sample and the signal captured by the MRI. Finding an adequate ratio between TR and TE is essential to enhance tissue contrast. By setting TE at its lowest possible value and only adjusting TR, the contrast of the sample can be easily and successfully adjusted.

NEX refers to the average number of times a RF pulse is applied to a sample and the signal received by the MRI. Increasing NEX helps improve the SNR but increases scan time.

FOV determines the area being imaged. It is important to set an FOV large enough to capture the sample and balance NEX.

The horizontal and vertical resolution, along with slice thickness, defines the voxel size within the area being scanned. Smaller values create a smaller voxel size, which is able to capture more structural details within the sample.

Regarding cross‐platform parameters, although we do not currently have access to additional preclinical low‐field MRI systems, the parameters outlined in this article should be broadly transferable. Minor adjustments may be required to achieve optimal performance on other preclinical low‐field MRI scanners. However, because preclinical low‐field MRI platforms generally share similar magnet designs and core hardware components, the overall workflow and imaging parameters described here should be applicable to other systems with only minimal modification.

### Troubleshooting

For troubleshooting suggestions, see Table 1.

**Table 1:** Troubleshooting Guide for High‐Quality Sample Imaging with a 1‐Tesla Compact MRI

Problem	Possible cause	Solution
Software crash	Excessive NEX	Increase the FOV
Poor contrast	Suboptimal TR/TE ratio	Incrementally increase TR by 10 ms
Poor SNR	Low‐resolution acquisition parameters	Increase NEX by 10
Coil calibration failure	Unsuccessful system calibration	Run a calibration protocol
Sequential coil calibration failure	Insufficient free protons within the FOV	Wrap the syringe with additional wet gauze
RF coil calibration failure	Unsuccessful system calibration	Run an RF calibration protocol using a phantom
Image motion artifact	Sample movement during the scan	Tighten the syringe by pushing the plunger upward to secure the sample position (while ensuring the sample is not overly compressed)

### Time Considerations

#### Basic Protocol 1

##### Obtain and prepare ex vivo tissue samples

Performing perfusion of one mouse with heparinized saline takes ∼10 min. Collecting organs takes ∼20 min, followed by a 24‐hr soak in 10% formalin. After soaking, organs can be transferred to 70% ethanol for storage until use.

##### Soak in contrast agent Gd‐DTPA

If used immediately, organs are quickly rinsed in PBS for 5 to 10 s, followed by a 24‐hr soak in Gd‐DTPA solution.

##### Embed sample in Fluorinert

Embedding a sample in Fluorinert takes approximately 5 to 7 min.

#### Alternate Protocol

Preparing the agarose samples takes ∼10 min.

#### Basic Protocol 2

The optimized scan time is ∼13 hr.

### Author Contributions

**Andrea Litwak**: Designed the research; performed the research; performed the image data analysis; drafted the manuscript; revised and proofread the manuscript. **Andrea Sarabia**: Designed the research; performed the research; performed the image data analysis; drafted the manuscript; revised and proofread the manuscript. **Mikayla Tamboline**: Designed the research; performed the image data analysis; supervised, advised on, and discussed data generated by Andrea Litwak and Andrea Sarabia; revised and proofread the manuscript. **Shumpei Mori**: Prepared and provided the human heart sample; performed the image data analysis; revised and proofread the manuscript. **Shili Xu**: Designed the research; performed the image data analysis; supervised, advised on, and discussed data generated by Andrea Litwak and Andrea Sarabia; revised and proofread the manuscript.

### Conflict of Interest

The authors declare no conflict of interest.

## Data Availability

The data, tools, and material (or their source) that support the protocols are available from the corresponding author upon reasonable request.
